# The Critical Exploration into Current Evidence behind the Role of the Nutritional Support in Adult Patients Who Undergo Haematogenic Stem Cell Transplantation

**DOI:** 10.3390/nu15163558

**Published:** 2023-08-11

**Authors:** Piotr Pawłowski, Paulina Pawłowska, Karolina Joanna Ziętara, Marzena Samardakiewicz

**Affiliations:** 1 Student Scientific Association at the Department of Psychology, Faculty of Medicine, Medical University of Lublin, 20-081 Lublin, Poland; pawlowskipiotr56@gmail.com; 2 The Critical Care Unit, The Royal Marsden Hospital, London SW3 6JJ, UK; paulina.pawlowska@rmh.nhs.uk; 3 Department of Psychology, Psychosocial Aspects of Medicine, Medical University of Lublin, 20-081 Lublin, Poland; marzena.samardakiewicz@umlub.pl

**Keywords:** nutritional support, nutrition, haematogenic stem cell transplantation, HSCT, haematooncology

## Abstract

Haematopoietic stem cell transplantation (HSCT) is a treatment option for many haematological conditions in patients of all ages. Nutritional support is important at each stage of treatment, but particular nutritional needs and dictated support occur during the preparatory (conditioning regimen) and post-transplant periods. Patients may require nutritional treatment by the enteral or parenteral route. The quantitative and qualitative composition of meals may change. Vitamin requirements, including vitamin D and vitamin C, might also be different. An adequately composed diet, adapted to the needs of the patient, may influence the occurrence of complications such as graft-versus-host disease (GvHD), gastrointestinal disorders, infections, and reduced survival time. Haematological diseases as well as transplantation can negatively affect the intestinal flora, with negative consequences in the form of mucosal inflammation and disorders of a functional nature. Currently, aspects related to nutrition are crucial in the care of patients after HSCT, and numerous studies, including randomized trials on these aspects, are being conducted. This study serves the critical analysis of current scientific evidence regarding nutritional support for patients after HSCT.

## 1. Introduction

Haematogenic stem cell transplantation (HSCT) is a potentially curative, intensive but also challenging approach to treatment for a variety of haematological malignancies [1,2,3,4,5]. However, multiple factors can potentially induce changes in metabolism and body compositions at the time of HSCT, including a high dose of systemic anticancer therapy (SACT), steroids, immunosuppressors, and total body radiation (TBI) which can lead to complications such as mucositis, nausea, vomiting, decreased indigestion, infections, or graft-versus-host disease (GvHD) [1,6,7,8,9,10,11,12,13,14,15]. Nutritional support prevents protein wasting, preserves the lean body mass and energy reserves, recontracts immunity, and diminishes the inflammatory response [15,16,17,18]. It was found that energy requirements after HSCT commonly increase by 30–50%. In addition, 10–50% of all patients suffering from haematological malignancies are already malnourished before the transplant [15,17,18]. Moreover, malnutrition and weight loss were identified with increased morbidity and non-relapsed mortality but also with decreased quality of life of adult patients undergoing HSCT [5,17,19].

Nutritional support and a holistic approach are required throughout the journey of post-transplant patients [17,19,20,21]. Adequate assessment, type of nutrition, and application of novel therapies may prevent malnutrition, further complications, poorer treatment outcomes, and even early death but also improved patients’ quality of life and sense of well-being. Professionals who provide direct care to very sick haematooncology patients with post-transplant complications experience the emergence of all the above statements when caring for this group of patients [22,23,24].

The aim of this review is critically analysing the available literature on the aspect of nutritional support provided for adult patients after HSCT and illustrates how current recommendations to improve patient care may be implemented in practice.

## 2. Material and Methods

The study used a focused non-systematic review of the available English-language literature. The time limit was set at the standard five-year period for medical science (2018–2023). Source material was from 2012–2018. In order to present a given subject in a holistic way and to present the dynamics of its development, the source materials of the selected records were analysed, and therefore relatively older studies from earlier years were included in the review. However, efforts were made to include the latest, innovative reports on the analyzed topic. Databases such as PubMed^®^, Scopus, Web of Science, and Google Scholar were searched. The main research question was “What is the role of different forms of nutritional support in adult patients undergoing haematopoietic stem cell transplantation”and keywords were selected accordingly: nutritional support, haematopoietic stem cell transplantation (HSCT), enteral nutrition, parenteral nutrition, vitamins, dietary supplements, and gut microflora.

Randomised clinical-control trials, and retrospective and prospective clinical studies, as well as systematic reviews and meta-analyses were eligible for analysis. The results of the database searches were initially analysed by title and abstract. In the next stage of the research process, two reviewers independently reviewed the full content of the accepted articles and, in the event of a divergence in the decision to include a publication, a discussion was held among all members of the research group until a resolution was reached and a consensus reached on a joint decision. The action described above was intended to ensure a high level of credibility.

## 3. Nutritional Assessment and Screening

Malnutrition has been proven to be a poor prognostic indicator for HSCT patients [19,25,26,27,28]. The risk of mortality might be twice as high in the HSCT when malnourished [15,27,29]. To prevent it, assembling adequate planning and support for the nutrition of patients who undergo HSCT, nutritional risk assessment, and screening are required [17,30,31]. The American Society for Parenteral and Enteral Nutrition (ASPEN) declares that nutritional treatment of all patients with haematooncological disorders should be initiated from the correct nutritional assessment [23].

Liu et al., 2016 carried out a cohort study, enrolling 170 patients from the HSCT centre in Pekin and examining different ways of evaluating the nutritional status of patients before and after receiving HSCT treatment. The study concludes that any nutritional support for this group of patients originates from timely and precise evaluation. The variation of nutritional assessment methods should be used in clinical practice and all patients with haematological malignancies must be treated with complexity regarding the nutritional status in all the stages of disease or treatment, including HSCT. Single-centred and non-randomisation characteristics remain weaknesses of this study [32].

Innovatively, Botti et al., 2015 focused on Italian HSCT centres that offer allogeneic HSCT (allo-HSCT) and autogenic HSCT (auto-HSCT) and demonstrated valuable differences between local guidelines and regular clinical practice. Noticeably, around 46% of hospitals did not have established protocols to evaluate the nutritional status of their patients. Despite that, most of the centres’ screening for malnutrition risk was performed. The survey form of the paper was found to be a major limitation [30].

There is no gold principle in assessing the state of nutrition of HSCT patients [12]. ASPEN and European Society for Clinical Nutrition and Metabolism (ESPEN) recommendations declare that all HSCT patients should be screened for malnutrition; however, there is no general agreement on how to assess malnutrition in this group of patients. Indicating contrast, the nutritional status of HSCT patients may be evaluated by MUST (Malnutrition Universal Screening Tool), NRS-2002 (Nutritional Risk Screening 2002), MNA-short Form (MNA-SF), or The Malnutrition Screening Tool [33,34,35,36,37]. Due to simple accessibility, NRS-2002 is strongly advised by ESPEN [38]. Along with nutritional status, the muscle atrophy should also be screened in a combination of two easily validated clinical tools—MUST and SARC-F (Strength, Assistance in walking, rising from a chair, Climb stairs, and Falls) [38]. According to Sagou et al., 2019, the nutritional risk index (NRI) is a useful tool to assess the nutritional status in allo-HSCT patients. That study, conducted in Japan, has shown that low pre-transplant NRI significant impacted the overall survival and nonrelapse mortality of those patients. Oppositely, high NRI was related to the higher incidence of grade II-IV acute and chronic GvHD [25].

An interesting point of view is the measurement of body compositions in patients undergoing HSCT as an indicator of nutritional status. Barban et al., 2020 stated that a useful tool for nutritional surveillance, determining under or overweight conditions in HSCT patients in Brazilian population patients, is anthropometric measurements [39]. Body structure should be consistently assessed in patients undergoing HSCT. The body weight, skin fold thickness, and arm and arm muscle diameter are measured, and BMI should be calculated [31,40,41]. However, there are many conditions and parameters which might present as a limitation for this type of nutritional assessment in haemato-oncological patients undergoing HSCT. It is important to remember that the presence of peripheral oedema, splenomegaly, ascites, or plural oedema may affect the body weight. This might differ as well due to fat accumulation due to corticosteroid therapy [42]. Another indicator of impaired nutritional status as well as an indicator of the risk factor for HSCT survival rate is skeletal muscle measurement. However, a specialist, high-precision body composition analysis device is required for this examination [43,44,45].

The study carried by Liu et al., 2013 in Pekin on a group of 99 leukaemic patients who were prepared for HSCT by analysing the appropriateness of using the NRS-2002 scale in this study group clearly states that this tool is only suitable for screening nutritional risk before HSCT. Beyond this, there was no significant difference in nutritional risk between men and women, and patients of less than 30 years old, not-full matched, recent (1–3 months) weight loss, reduced food intake within a week, or BMI < 18.5 were more likely to have nutritional risk. This may suggest that although there is no age restriction for the NRS-2002 in patients under 30 years of age, nutritional risk should be assessed with a different tool. However, the study group was small, which is a major limitation of the described study [46].

Lui et al. in 2012 in China conducted a comparative study of three different methods of (NRS-2002, MNA, Subjective Global Assessment (SGA)) assessing the nutritional status of 50 leukaemia patients before HSCT. Analysing the results, the authors concluded that nutritional risk scores differed significantly. In the case of the SGA, only one case (2%) was loaded or moderately malnourished, which is significantly different from the results in the other two tools (NRS-2002—13 subjects (26%), MNA—12 subjects (24%). Paired x2 test results showed that the difference between NRS2002 and MNA was statistically significant (x2 = 13.64, *p* < 0.05), Kappa test results showed that they were consistent between NRS-2002 and MNA (Kappa = 0.62, *p* < 0.05). Therefore, the authors suggest that in patients being prepared for HSCT, the NRS-2002 should be the first choice of nutritional assessment, and that using it concurrently with the MNA may positively affect the accuracy and comprehensiveness of the assessment [47].

The importance of adequate nutritional assessment for patients pre- and post-HSCT is unquestionable. The evidence behind the patient’s screening is strong. There are multiple current studies which confirm the impact of malnutrition on pre- and post-transplant patient’s overall survival rate and also on nonrelapse mortality [15,18,23,25,26,48,49,50]. The assessment of the nutritional status of patients after HSCT is controversial, due to the high impact of inadequate screening and further assessments on the initiation of nutritional treatment. Above and beyond this, there is no consensus on unambiguous evaluation of these tests, indicating clear cut-off points initiating in-depth assessment. An abnormal screening result also does not provide satisfactory data for the design of individualised, patient-specific nutritional pathways [27,38]. There is also a lack of multicentred studies regarding nutritional screening in HSCT which might be a consequence of the absence of standardized protocols across the centres [30]. A summary of the tools potentially used to assess the nutritional status of patients before and after HSCT, along with their description, is presented in Table 1.

## 4. Enteral versus Parenteral Nutrition

For HSCT patients who are unable to maintain their oral route of nutrition due to gastrointestinal system failure, the nutritional support is indicated as either enteral nutrition (EN) or parenteral nutrition (PN) [38,56,57,58]. According to ASPEN based on the theory, the benefit of EN is to maintain close connections between intraepithelial cells, creating blood flow and insisting that tropic endogenous agents (e.g., gastrin) are principal in the underlying stability of the GI system [23]. The use and benefits of PN compared to EN appear to be controversial in the literature. PN allows the administration of nutrition at the designed level when the patient suffers from GI complications and dysfunctions related to HSCT. It is also not correlated with GI disturbances. However, many complications are related to PN compared to EN. The current recommendations in favour of EN over PN are mainly based on pathophysiological deliberations and evidence from smaller studies claiming that PN is related to higher risks of metabolic and infectious complications [26,49,59].

The diagnosis of haematological malignancy and HSCT procedure are fundamental factors affecting patients’ nutritional status. Muscle protein depletion, metabolic derangements, and malnutrition are frequently dominant aspects caused by the disease itself or given treatment (intensive SACT, TBI, HSCT) [26,49]. Low BMI and cachexia impact the overall survival of this group of patients. To prevent further complications related to malnutrition, ESPEN recommends that an oral route of feeding be maintained unless there is severe mucositis, vomiting, malabsorption, diarrhoea, or uncompromising symptoms of GI GvHD. An early EN implementation is approved unless contradictory. Due to the high risk of infectious complications, PN is advised as a last option [27,38].

The current systemic review on nutritional support in HSCT emphasizes the limited available evidence to direct decisions in this area. Despite the limitations, Andersen et al., 2019 in Australia carried out a randomised, single-centre trial regarding the tolerability of proactive EN post-allo-HSCT based on current ESPEN recommendations [60]. The study concluded that EN should be used as a first-line therapy for patients post-allo-HSCT; it also highlighted that the use of proactive EN would decrease of application of PN, which is associated with higher costs and risks. The serious weakness of this study is the potentially small size of the control group of patients involved. Similar concerns exist for the large, multicentred, and randomised NEPH. A study conducted in France validates the same conclusions regarding the implementation of EN. It verified that the enteral route of nutrition might directly decrease the risk of immunological and infectious events in allo-HSCT patients, as well as diminish early transplant-related morbidity and mortality. Compared to Andersen et al., 2019, the control group remained twice as large [60,61]. An identical comparison came from Guièze et al., 2014, investigating the impact of EN versus PN on the early outcome of French patients who required nutritional support post-allo-HSCT in one of the French transplant centres. The study confirmed the predominance of EN compared to PN. EN was associated with much lower risks of infections and without the increase in the occurrence of GvHD [62].

There is strong evidence behind an early implementation and the benefits of EN compared to PN; however, collected data and clinical experience might suggest that EN via nasogastric tube (NGT) feeding (or any other enteral feeding device) is still avoided by health care professionals and HSCT patients themselves [15,26,49,62]. A possible reason for this might be the easier associability to central venous catheters, which are frequently inserted before HSCT for intensive SACT. Additionally, it is noticeable that there is a lack of empirical and holistic research regarding why current recommendations are not implemented in the form of policies or protocols in HSCT populations when nutritional support is required [61,62,63,64].

Another important key point to mention is that since 2009, ASPEN has not updated its guidelines regarding nutritional support therapy during anticancer treatment and in HSCT, which might cause restrictions and limitations to using them in clinical practice. On the other hand, it is important to acknowledge that there are not many recent studies carried out in the last 14 years despite the fact that patients after HSCT and their GI complications are significantly better understood and managed currently than at that time [65]. Surprisingly, a large meta-analysis carried out by Chow et al., 2020 states that further studies regarding the advantageous use of EN over PN in cancer patients might be unnecessary as they may add potentially little value to the existing recommendations which have been known for decades [66]. A summary of enteral and parenteral nutrition is provided in Table 2.

## 5. Neutropenic Diet

In HSCT patients after receiving multimodal treatment required in haematological malignancies, including SACT, TBI, and additional doses of immunosuppressants and corticosteroids, the suppression of bone marrow does appear [69]. This can cause a common and suspected side effect—neutropenia. A state of low neutrophils within blood post-transplant is related to a higher risk of infections but also to possible life-threatening sepsis. Numerous prophylactic interventions are taken to control the risk of neutropenic sepsis, and one of them is a neutropenic diet [33,69,70,71,72].

Traditionally, all patients who suffer from neutropenia were prescribed a neutropenic diet to reduce the risk of exposure to foodborne microorganisms [73]. Although multiple studies in the past have examined infection risks and diet, the prophylactic advantage of a low-microbial diet against infections has been not confirmed [33]. By comparing the neutropenic diet and non-neutropenic diet in patients post-transplant, Lassiter et al., 2015, in North Carolina confirmed that there is no significant difference between the infection rate or nutritional status in those patients [26]. The neutropenic diet did not provide preventable effects in the two groups of HSCT patients involved. Ramamoorthy et al., 2019 carried out a systemic review of the available literature regarding the benefits of a neutropenic diet to patients and reported that there is a lack of evidence regarding the advantages of neutropenic diet strategies; it is discernible that various approaches are required [69]. Similarly, Ma et al., 2022 in their meta-analysis, found that implementation of a neutropenic diet cannot diminish the risk of infection or even mortality [74].

There is a lack of standardized protocols regarding diet in neutropenia and the controversies between the centres are observable [33]. Baumgartner et al., 2017 in their systematic review confirmed that most centres offering angio-HSCT did not follow any kind of neutropenic diet, while those offering allo-HSCT applied neutropenic diets on their patients had recognisable differences in its types [26]. A national survey carried out by Carr and Halliday in 2015 revealed valuable insight into the use of a neutropenic diet and recommendations regarding it by oncology and haematology dieticians across the UK. The survey revelated that around 67% of dieticians who participated recommended the use of a neutropenic diet for their patients. Further, higher-quality evidence regarding this matter is needed [75].

**Figure 1:**
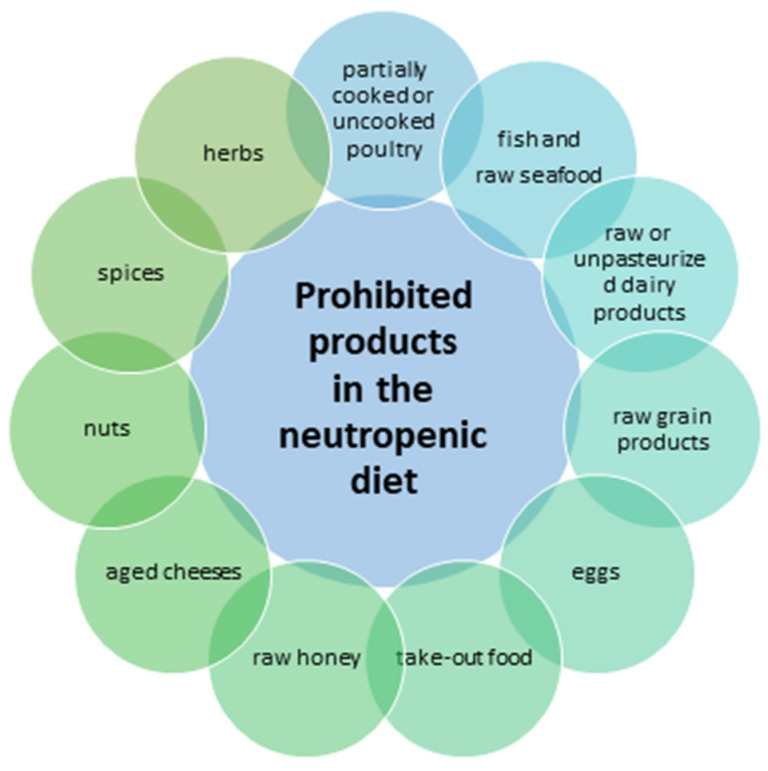
Prohibited products in the neutropenic diet (based on [69]).

ASPEN recommends supporting neutropenic patients with nutritional advice on foods that might put them at risk of infection along with safe food preparation and consumption [23]. However, current recommendations commenced by ESPEN state that there is not enough insufficient, consistent, and good-quality data related to the use of low-bacterial diets in HSCT patients [38]. Instead of a restrictive neutropenic diet, the key here would be the educational aspect of safe food handling for HSCT, neutropenic patients by instructing them on the safe preparation, separation, cleaning, and storage of food. It has been shown that this might have a positive impact on their enhanced oral intake, high satisfaction from consumed food, and possible improved quality of life. However, more evidence is needed to investigate this matter in older populations of patients post-transplant [24,75,76]. Prohibited products during the use of a neutropenic diet as an element of infection prophylaxis are listed in Figure 1.

## 6. Glutamine

Glutamine is an amino acid, glutamic acid amide, and has been shown to be a source of carbon and nitrogen for cellular biosynthetic fuel, mainly for enterocytes and lymphoid tissue. During many diseases (including cancer) and stress, the demand for L-glutamine increases, with a consequent drop in its concentration by up to 50% from baseline. The scientific evidence on the clear benefits of enteral or parenteral administration of this amino acid is inconclusive [77,78].

A randomized, double-blind, controlled study conducted by Ziegler et al. in the 1990s, in Boston, Massachusetts suggested that the use of glutamine at a dose of 0.57 g/kg b.w./day with PN has a positive effect on nitrogen balance and reduces the risk of infection, thus shortening the overall length of hospitalization [79]. A metanalysis by Crother et al. that included 17 randomised clinical-control trials involving patients after HSCT found that this amino acid administered in oral form reduces mucositis and will reduce the risk of GvHD, while in intravenous form it reduces the incidence of clinical infections. However, the effect of glutamine on overall mortality after HSCT was not demonstrated [80].

The most recent retrospective observational study analysing glutamine administration in post-HSCT patients that the authors were able to access is from 2023. It was conducted in Spain by Domiguez. It showed no significant difference in the incidence of mucositis between patients in the glutamine and non-glutamine groups, but diarrhoea was more frequent in the latter group. It is worth noting that, according to this study, infections were more frequent in patients receiving glutamine, and the length of hospital stay was also significantly longer in this group. No differences in mortality were shown. In their conclusion, the authors of this study do not recommend the routine use of glutamine in patients after HSCT [81]. A systematic review of retrospective and prospective studies with a control group by Sayles et al. demonstrated the efficacy of oral glutamine in 11 of the 15 manuscripts analysed, particularly in the context of mucositis. However, for placebo-controlled studies, only two out of six studies confirmed this relationship [82]. Data have also emerged highlighting the association of glutamine with higher rates of tumour recurrence in patients after HSCT [83]. Due to the high heterogeneity of research data, current recommendations emphasise that it is not possible to unequivocally recommend the use of glutamine in patients after HSCT (ESPEN) [38].

## 7. Polyunsaturated Fatty Acids

Polyunsaturated fatty acids (PUFAs) are a group of compounds containing at least 18 carbon atoms and two double bonds in their chemical molecular structure. Examples of such compounds are α-linolenic acid, 18:3 (ω-3), arachidonic acid, 20:4 (ω-6), docosahexaenoic acid, 22:6 (ω-3) (DHA), and eicosapentaenoic acid 20:5 (ω-3) (EPA) [84,85,86,87]. The omega-6 PUFAs exhibit pro-inflammatory effects by stimulating the production of specific cytokines, while the omega-3 PUFAs are widely regarded as those showing anti-inflammatory and immunomodulatory properties [21]. The latter belong to a group of macromolecules with well-established evidence of positive impact when used in patients with haematological proliferative diseases, including in patients post-HSCT [84,85,86,87].

A small study by Takatsuka et al. in 2001 in Japan analysed the impact of EPA on prognosis and complications associated with bone marrow transplantation. Sixteen allogeneic transplant patients from unrelated individuals were enrolled in the study, seven of whom received 1.8 g/day of EPA orally, from 3 weeks before HCT until approximately day 180 after transplantation. Conclusions from the study suggest that the use of this macromolecule has a positive effect on the reduction of pro-inflammatory cytokines such as leukotriene B(4), thromboxane A(2), and prostaglandin I(2); in addition, tumour necrosis factor-alpha, interferon-gamma, and interleukin-10 were reduced. Over and above, the EPA-taking group had a lower severity of GvHD compared to the other subjects [88]. Given the sample size and the single-centre limitation, the results should be approached with caution as their strength is weak.

A 2016 randomised, double-blind, clinical-control study by Baena-Gomes et al. in Spain analysed the effect of a fat emulsion of omega-3 acids administered with PN on the intensity of the subjects’ immune response (IL-1β, IL-2, IL-6, IL-8, IL-10 levels). The test sample was given fish oil, while the control sample was given soybean oil. Blood for analysis was collected at time point 0, day 10, and day 21. The first-time interval showed no significant differences in the levels of pro-inflammatory cytokines, but these markers decreased significantly after the 21-day period. These data demonstrate that short-term administration of Omega-3 has no benefit, but its long-term administration shows immunomodulatory and anti-inflammatory properties [89]. The inclusion of omega-3 PUFAs as a component of PN appears to be particularly beneficial in patients with intestinal GvHD, patients with hypertriglycerinaemia (to maintain a normal lipid ratio in PN) [58].

Numerous lines of evidence suggest beneficial effects of omega-3 PUFAs [90,91,92,93], but no international working group has decided to explicitly indicate their routine use in patients after HSCT. They are suggested for use in cases undergoing chemotherapy and at risk of weight loss or malnutrition. As several positive studies were published in the last few years which showed nutritional benefits, a plausible biological rationale, only mild side effects, and no convincingly serious safety issues, a weak recommendation was produced for the use of fish oil and a long-lasting one for N-3 fatty acid chain [27,38].

## 8. Vitamin D

There is a notable lack of research on the micronutrient intake of HSCT recipients, particularly about their risk for malnutrition and micronutrient deficiencies during the acute phase of transplant [94]. This is concerning given the well-established link between malnutrition and negative health outcomes in transplant patients [95]. However, Vitamin D (VD) has been shown to play a crucial job in the pre- and post-HSCT process. Calcitriol, which is the active form of VD, is a vital regulator in haematopoiesis. It binds to vitamin D receptors and plays an important function in immunity [96]. Caballero-Velázquez et al., 2016 provides an interesting insight into the role of VD on the incidence of chronic GvHD (cGvHD) among HSCT patients. In Spain 150 patients were involved and divided into three different cohorts (50 patients each): control group (not received VD), low dose and high dose of VD Patients received VD from day 5 to 3 months after allo-HSCT. It was noticed that the incidence of grade II-IV cGvHD was twice as low in those patients who received high doses of VD, without a significant increase in the occurrence of infections or relapse. However, there was no significant difference in the incidence of aGvHD. Furthermore, the effects of VD on immune response post-allo-HSCT displayed lower levels of CD40L in T cells and a lower number of B cells which might play a central role in the incidence of cGvHD. Overall, current studies with a bigger population of HSCT patients, focusing on the influence of VD, on immunity are required [97].

Similarly, Daloğlu et al., 2023, in Turkey investigated the relationship between pre-HSCT plasma vitamin D levels and acute form of GvHD in adults undergoing HSCT. The study found that patients with lower pre-transplant VD levels had a remarkably higher rate of acute GvHD (aGvHD) compared to those with higher levels. Their findings suggest that maintaining adequate pre-transplant vitamin D levels might be valuable in reducing the risk of aGVHD in adult HSCT patients. However, there was no significant association between vitamin D levels and overall survival or non-relapse mortality in this group of patients [98].

Hong et al., 2020 carried out a meta-analysis regarding the role of VD in HSCT patients. They confirmed the potential benefits of VD, its role in immune function and the possibility of reducing the risk of GvHD. However, they also observed that the evidence remains varied, and that further research would be needed to fully understand the relationship between VD and HSCT patients’ outcomes. Additionally, the authors highlighted that many of the studies included in their review were observational, which makes it difficult to demonstrate valuable data [99].

The maintenance of adequate levels of VD among HSCT is important as it may help optimize immune function and improve patient outcomes [95,96]. Overall, VD plays a significant role in haematopoiesis and immunity and might play a vital role in preventing aGvHD, but further research is needed to fully understand and view its mechanisms of action and potential therapeutic benefits pre- and post-transplantation, especially considering that the majority of the current studies are focused on the paediatric population of patients [99].

## 9. Vitamin C

Vitamin C (ascorbate) is a water-soluble vitamin and its main function is to act as an antioxidant. It also influences the synthesis of connective tissue-building collagen, corticosteroids, and some neurotransmitters, and is important in iron absorption and haematopoiesis [100,101,102,103]. Much evidence suggests the involvement of vitamin C in immune enhancement processes [104]. The human body does not have the capacity to synthesise it on its own—it must be supplied with food [101].

Post-radical chemotherapy patients being prepared for HSCT have an increased risk of infection due to neutropenia, so many researchers have highlighted the potential for vitamin C to be used in strengthening the infectious immune response. The depletion of stores of this micronutrient has also been confirmed by studies [105]. A study by Carr et al. in 2020 in New Zealand analysed the status of vitamin C, C-reactive protein, and oxidative stress (protein carbonyls and thiobarbituric acid reactive substances) concentrations in 38 patients who underwent conditioning chemotherapy and HSCT (at interval 0, 1, 2, 4 weeks after transplantation). The lowest mean vitamin C concentrations (recorded at week 2) corresponded to the highest mean C-reactive protein values and the lowest mean neutrophil count. On this basis, it can be concluded that neutropenic fever, increased inflammation, and oxidative stress coincide with impaired vitamin C status [105].

A prospective study by Urbalejo-Ceniceros et al. (Mexico) of the administration of high doses of vitamin C (20 g/day 1–10 day, 1 g/day 11–100 days) in 24 patients after HSCT showed that NK and CD3+ lymphocytes in the group treated with this micronutrient showed an increase, over and above a lower incidence of infection. No serious side effects of this treatment were demonstrated. The authors of the referenced study therefore suggest that high-dose vitamin C supplementation is an effective and safe therapeutic option to reduce the frequency of infections and enhance immune reconstitution after HSCT [106].

A randomised double-blind clinical-control study negating the efficacy of high-dose vitamin C administration in patients after HSCT has also emerged. van Gorkom et al., in Netherlands enrolled 44 patients, 21 of whom received vitamin C. The results only confirmed a lower incidence of bacteraemia in the group that received this micronutrient. No significant difference was observed between the groups in neutrophil recovery time, hospitalisation time, incidence of neutropenic fever or 3-month overall survival. The authors of this study explicitly do not recommend vitamin C supplementation in this treatment group [104].

## 10. Gut Microbiome—Probiotics, Prebiotics, Faecal Microbiota Transplantation

Research into disorders of the naturalised intestinal flora in haematological malignancy patients has intensified in recent years. Post-HSCT patients experience changes in the gastrointestinal microbiome because of pharmacotherapy, stress, functional and intestinal dysfunction, and inflammation of their mucosa [107,108,109]. The role of the microbiome on the outcome of allo-HSCT has also been demonstrated: the dominance of enterococci in the gut correlates with the spectrum of antibiotics administered to patients and leads to reduced survival rates [109]. A drastic decrease in gut microbial biodiversity during conditioning and HSCT is associated with a pro-inflammatory immune response and consequently an increased risk of transplant-related complications such as aGvHD or increased mortality [110].

**Figure 2:**
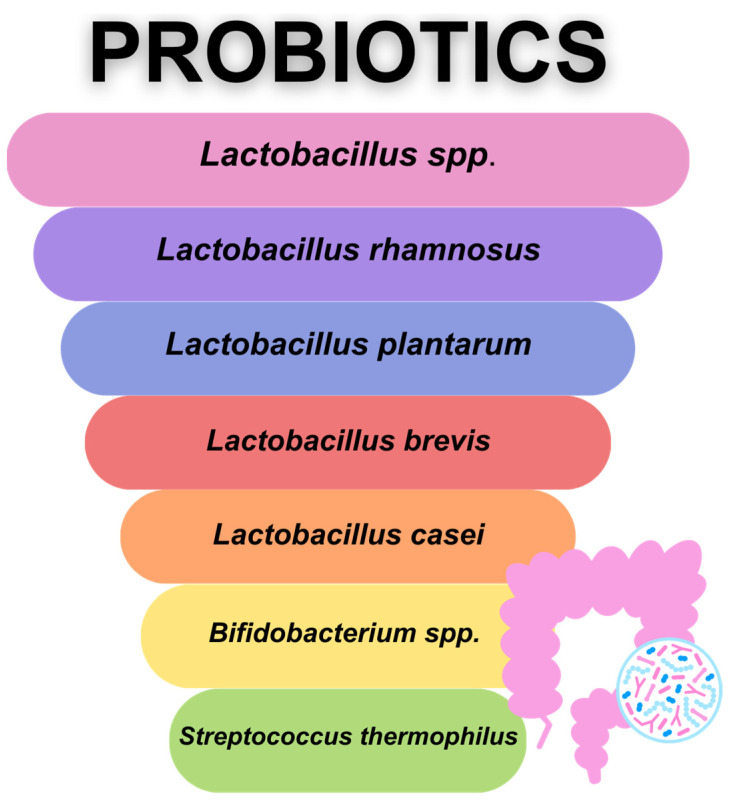
Bacterial strains used in probiotic therapy (based on [112,122,123,124,125,126,127,128,129,130,131,132,133]).

Probiotics are living organisms used to rebuild the gut microbiome. For patients after HSCT, preclinical as well as clinical studies have produced inconsistent results [110]. In one study in a mouse model, administration of *Lactobacillus rhamnosus* GG reduced the incidence of aGvHD [111]; however, the randomised clinical-control study by Gorshein et al. (New Jersey) did not confirm this relationship [112]. Disruption of the intestinal mucosal barrier superimposed on reduced immunocompetence and immunity can result in bacteraemia as a complication of probiotic therapy. A retrospective analysis of 3796 post-HSCT patients in Seattle, Washington receiving probiotic supplementation showed that 19 (0.5%) patients developed bloodstream infections (BSI), mainly due to *Lactobacillus* spp. [113]. Figure 2 includes the names of the bacterial strains used during HSCT.

Prebiotics include chemical compounds that are the fermentation substrate of intestinal bacteria. These include, for example, starches, fructooligosaccharides and galactooligosaccharides. Retrospective studies mainly indicate benefits in terms of mucosal inflammation, the occurrence of diarrhoea, and a lower risk of bacteraemia compared to probiotics. However, no reduction in the incidence of GvHD was observed [110,111,112,113,114].

One of the most recent randomised double-blind studies comparing the administration of a symbiotic (a combination preparation of a prebiotic and a probiotic) was conducted in Tehran. Of 40 allo-HSCT patients, 20 received a daily symbiotic 21 days prior to transplantation (days −21 to day 0); the remaining participants were untreated controls. The incidence of severe (grade III/IV) aGVHD in the study group was 0% (0 out of 20 patients), while in the group without the symbiotic it was 25% (five out of 20 patients). Above this, the overall survival rate in the study group was higher than in the control group, but the difference was not statistically significant. The study authors suggest that taking symbiotic before and during the conditioning regimen of allo-HSCT patients may lead to a reduction in the incidence and severity of aGVHD [115].

The possibility of faecal microbiota transplantation (FMT) is also one way of influencing the gut microbiome. This type of therapy is currently experimental, except in cases where the main reason for performing this procedure is the patient’s Clostridium difficile infection (CDI) and multidrug-resistant bacteria [116,117,118]. The efficacy of such management of intestinal dysbiosis in patients after HSCT is confirmed by Webb et al. describing the preliminary results of a programme conducted at a US centre in 2016. Seven patients were studied; diarrhoea improved in all patients after FMT, six of the patients had no relapse, and one patient had a relapse on day 156 after FMT after taking an oral antibiotic and required repeat FMT, followed by no relapse. Despite the small study group, one can venture to conclude that with careful donor selection and laboratory screening, microbiota transplantation appears to be a safe and effective therapy for CDI in patients after HSCT and may have additional benefits [117].

Several studies have suggested the efficacy of FTM for the treatment of aGvHD, mainly the steroid-resistant form, but according to the available data, mortality was not reduced; therefore, the efficacy of such management for the prevention or early intervention of aGVHD was analysed and higher survival results were obtained [119,120]. The strength of this evidence is relatively low, due to the small size of the study groups and the pilot nature of the study. Unfortunately, there is a lack of clinical-control randomised trials on the use of this procedure. A prospective, open-label, multicentre, randomised phase II parallel-group clinical trial is currently underway, designed to evaluate the effect of FMT on toxicity in patients treated with myeloablative allo-HSCT for haematological malignancy. The project envisages the inclusion of 60 male and female patients aged 18 years or older and is led by the French team of Dougé et al. [121].

## 11. Summary

This literature review investigated most issues highlighted by researchers’ trends in nutritional status and support for HSCT patients. These groups of patients face several symptoms and complications that might have a major impact on their food intake, leading to a consequential risk of malnutrition. Therefore, interventions such as nutritional screening and assessment to enhance the nutritional status of HSCT patients are beneficial to improve the quality of their life as well as clinical outcomes [19,25,26].

Nutrition remains one of the few supportive therapies for HSCT. Several studies and official recommendations, involving the population of HSCT patients, confirm the feasibility and safety of EN over PN [26,27,38,49,62]. There is no strong evidence behind the implementation of a neutropenic diet in post-HSCT patients while facing neutropenia. It has been proven that the educational aspect of safe food handling for HSCT, neutropenic patients might have a greater impact comparting to a very restrictive, neutropenic diet [76,134]. The impact of maintenance of adequate levels of VD among HSCT is important as it may help optimize immune function and improve patient outcomes [32,33]. Overall, VD plays a significant role in haematopoiesis and immunity and might play a vital role in preventing aGvHD in post-HSCT patients [99]. The use of ascorbate in these patients produces relatively different results [104,105,106]. The intestinal flora plays an important role in prognosis, especially in the context of GvHD [109,111,112,115,120]. To better understand the impact of nutritional support, type of diet, or nutrition in high-risk HSCT patients, larger-sized, multicentre, randomised trials as well as more robust recommendations are greatly required to diminish variations of the particle across partitioners and centres.

The authors of this study would like to suggest that service improvement might be the development of an educational program for staff working on the unit who look after critically ill patients post-HSCT. The aim of this project would be the improvement of knowledge and skills of staff, regarding the management and nutritional support of HSCT patients, leading to improvement of patient outcomes, experience, and quality of life.

## Figures and Tables

**Table 1:** Summary of the tools potentially used to assess the nutritional status of patients before and after HSCT (based on [39,46,47,51,52,53,54,55]).

No.	Name of the Screening Tool	Description of the Screening Tool
1.	Nutritional Risk Screening 2002 (NRS-2002)	Patients are scored on each of two comorbidities (1) nutritional deterioration and (2) disease severity, according to whether they are absent, mild, moderate, or severe, resulting in a total score of 0–6. Patients with a total score ≥ 3 are classified as being at nutritional risk. Malnutrition was estimated using three variables used in most screening tools: BMI, percentage of recent weight loss, and change in food intake. The variable of age was also considered; when the assessed exceeds 70 years of age, they are assigned 1 additional point.
2.	Malnutrition Universal Screening Tool (MUST)	It is a five-step tool using three independent criteria to determine overall malnutrition risk: current weight status using BMI, unintentional weight loss, and acute disease effect that has induced a phase of nil per os for >5 days. Each variable can be scored on a three-point scale as 0, 1, or 2. The overall risk of malnutrition is set as low (score = 0), medium (score = 1), or high (score > 2). Together, these three criteria are better predictors than each individually.
3.	Malnutrition Screening Tool (MST)	This tool consists of two questions about recent unintentional weight loss and reduced appetite resulting in a reduction in the volume of meals consumed. The first is scored on a 4-point scale according to the number of pounds lost; a positive answer to the second results in a score of 1. The total score ranks from 0 to 5, with patients considered at risk of malnutrition if they score ≥ 2.
4.	Subjective Global Assessment (SGA)	It consists of the patient’s medical history (weight loss, food intake, gastrointestinal symptoms, and functional capacity) and physical examination (subcutaneous fat, muscle atrophy, and oedema). Patients are assessed as well-nourished (A), moderately malnourished (B), or severely malnourished (C). Typically used by clinicians as part of a comprehensive nutrition assessment.

**Table 2:** Summary of enteral and parenteral nutrition (based on [48,62,66,67,68]).

Enteral Nutrition		Parental Nutrition	
Advantages	Flaws	Advantages	Flaws
(1) The possibility of providing a complete feed. (2) May help to maintain the function and integrity of the gut barrier. (3) Increases IgA class immunoglobulin levels, which may reduce the frequency of respiratory infections. (4) Reduces the incidence of aGvHD grade III to IV (mainly intestinal forms). (5) Trophic effect on enterocytes. (6) Maintain a healthy gut mucosal and high gut microbiota diversity.	(1) Not to be used in persons with impaired gastrointestinal function. (2) Difficulties in obtaining gastric/enteric access. (3) Intolerance and side effects may occur (e.g., nausea and vomiting, non-occlusive bowel necrosis). (4) Retention of gastric contents promotes bacterial colonisation and increases the risk of ventilator-associated pneumonia. (5) Can be disturbed by patient care and diagnostic interventions, particularly among people receiving respiratory support.	(1) Provides a fully balanced diet, strictly controlled in terms of quality and quantity. (2) Ease of administration.	(1) Non-physiological route (bypasses the entire digestive tract and portal system). (2) The need for vascular access (complications). (3) Increases the risk of overfeeding. (4) More frequent disturbances of the economy (mainly hyperglycaemia). (5) Increased risk of infection (mainly catheter-related bloodstream). (6) High risk of distant complications, e.g., Essential Fatty Acid Deficiency (EFAD), protein losing enteropathy. (7) Intravenous fat emulsion can induce hyperactivation of the monocyte and macrophage system, leading to reduced platelet engraftment. (8) Reduced intestinal transit with subsequent gut microbiota dysbiosis and mucosal atrophy.

## Data Availability

Not applicable.
